# A Novel Clinical Six-Flavoprotein-Gene Signature Predicts Prognosis in Esophageal Squamous Cell Carcinoma

**DOI:** 10.1155/2019/3869825

**Published:** 2019-10-30

**Authors:** Liu Peng, Jin-Cheng Guo, Lin Long, Feng Pan, Jian-Mei Zhao, Li-Yan Xu, En-Min Li

**Affiliations:** ^1^The Key Laboratory of Molecular Biology for High Cancer Incidence Coastal Chaoshan Area, Shantou University Medical College, Shantou 515041, Guangdong, China; ^2^Department of Biochemistry and Molecular Biology, Shantou University Medical College, Shantou 515041, Guangdong, China; ^3^Institute of Oncologic Pathology, Shantou University Medical College, Shantou 515041, Guangdong, China

## Abstract

Flavoproteins and their interacting proteins play important roles in mitochondrial electron transport, fatty acid degradation, and redox regulation. However, their clinical significance and function in esophageal squamous cell carcinoma (ESCC) are little known. Here, using survival analysis and machine learning, we mined 179 patient expression profiles with ESCC in GSE53625 from the Gene Expression Omnibus (GEO) database and constructed a signature consisting of two flavoprotein genes (GPD2 and PYROXD2) and four flavoprotein interacting protein genes (CTTN, GGH, SRC, and SYNJ2BP). Kaplan–Meier analysis revealed the signature was significantly associated with the survival of ESCC patients (mean survival time: 26.77 months in the high-risk group vs. 54.97 months in the low-risk group, *P* < 0.001, *n* = 179), and time-dependent ROC analysis demonstrated that the six-gene signature had good predictive ability for six-year survival for ESCC (AUC = 0.86, 95% CI: 0.81–0.90). We then validated its prediction performance in an independent set by RT-PCR (mean survival: 15.73 months in the high-risk group vs. 21.1 months in the low-risk group, *P*=0.032, *n* = 121). Furthermore, RNAi-mediated knockdown of genes in the flavoprotein signature led to decreased proliferation and migration of ESCC cells. Taken together, CTTN, GGH, GPD2, PYROXD2, SRC, and SYNJ2BP have an important clinical significance for prognosis of ESCC patients, suggesting they are efficient prognostic markers and potential targets for ESCC therapy.

## 1. Introduction

Esophageal cancer has been ranked as the fifth most malignant disease, and it is the fourth leading cause of cancer death in China [1]. There are two main histological types of esophageal cancer: adenocarcinoma and squamous cell carcinoma [2]. Esophageal squamous cell carcinoma (ESCC) is the most common histologic type of esophageal cancer, accounting for approximately 90% of esophageal cancer tumors in China. Despite the advance in diagnosis, prognosis, and treatment, the early diagnosis for ESCC is poor, with a 5-year overall survival rate less than 20% [3]. It is widely believed that the occurrence and development of ESCC depends on alteration of multiple factors, multiple stages, and multiple genes, making it very likely that both genetic and environmental factors contribute to this disease [4]. Further understanding of the molecular mechanism of ESCC and screening of more efficient clinical markers are crucial for the early diagnosis and improvement in prognosis of ESCC patients.

Riboflavin (vitamin B2) is an essential micronutrient for normal cellular function. Riboflavin is the precursor of both flavin adenine dinucleotide (FAD) and flavin mononucleotide (FMN), which play key roles in cell development and growth. Riboflavin participates in intermediary metabolism and is required for many metabolic reactions, such as mitochondrial electron transport, fatty acid degradation, and redox regulation [5, 6]. An increasing number of researchers have found a positive correlation between riboflavin deficiency and cancer risk. Bassett et al. found that a higher intake of riboflavin is associated with decreased risk of breast cancer, and the same phenomenon has been observed for gastric, colorectal, and lung cancers [7–10]. Throughout recent years, there has been much research focused on the relationship between riboflavin and esophageal cancer. Siassi et al. suggested that riboflavin deficiency of residents is higher in areas with a high incidence of ESCC [11]. Our previous studies have shown that low plasma riboflavin levels are significantly associated with high risk and poor prognosis of ESCC patients, while after repletion of riboflavin can improve prognosis [4]. The above studies show that riboflavin deficiency is common in high-incidence areas of ESCC, suggesting that riboflavin dysfunction may be present in ESCC patients.

Deficiency of riboflavin can directly lead to the abnormal function of flavoproteins. Flavoproteins are a class of enzymes that catalyze a wide range of redox reactions through a variety of chemical mechanisms. This kind of protein must contain noncovalently bound FAD or FMN as a cofactor. Warburg et al. isolated the first flavoprotein from yeast (flavin phosphoric acid was its prosthetic group), and Banga et al. observed the presence of flavin in muscle tissue. So far, more than 160 kinds of flavoproteins have been isolated and characterized [12]. One of the most important characteristics of flavoproteins is the wide range of the catalytic reactions they performed, such as typical redox catalysis, DNA damage repair, or activation of dioxygen [13]. A number of recent studies indicate that abnormal expression of flavoproteins and their interacting proteins lead to a variety of clinical abnormalities, which range from degenerative changes in the skin lesions, to the growth retardation, or nervous system and peripheral neuropathy; it also affects the proliferation and mobility of various cancer cells [14–16]. However, the clinical significance and function of flavoproteins and their interacting proteins in ESCC still need to be evaluated. In this study, we obtained the expression level of flavoproteins and their interactors by re-annotating gene microarrays through analysis of the data and screened out a flavoprotein-related signature that can accurately predict overall survival of ESCC patients.

## 2. Materials and Methods

### 2.1. Flavoproteins and Their Interacting Proteins

We selected flavoproteins from the UniProt database (http://www.uniprot.org/) and identified their interacting proteins from the Human Protein Reference Database (http://www.hprd.org/) and BioGRID (https://thebiogrid.org/) database [17].

### 2.2. GEO Cancer mRNA Expression Data

The mRNA expression data and corresponding clinical data of 179 ESCC patients (GSE53625) were obtained from the GEO database (https://www.ncbi.nlm.nih.gov/geo/). We used this dataset as a training set [18] to analyze the correlation of flavoproteins and their interacting protein and the relationship between flavoproteins and the survival of ESCC patients. Probe re-annotation pipeline was performed as previously described [17, 19]. The probes which were perfectly matched to a transcript were retained. And the probes which targeted both noncoding transcripts and coding cDNA sequences were removed. The gene expression values were log2 transformed for all subsequent analysis.

### 2.3. Construction of a Weighted Overall Survival (OS) Predictive Score Algorithm

Firstly, we selected survival-related flavoproteins and their interacting genes by univariate Cox proportional hazards analysis (*P* < 0.05) and then used the random survival forests variable hunting (RSFVH) algorithm to filter out genes until 10 genes [18]. Subsequently, we developed a model to estimate prognosis risk as follows [18, 20, 21]:

(1) $$\begin{array}{c} \text{risk score }\left(\text{RS}\right)=\overset{N}{\underset{i=1}{\sum }}\left(\beta \;*\text{ EXP}\right), \end{array}$$

where *N* is the number of prognostic flavoproteins and their interacting genes, EXP is the gene's fold change value of expression, and *β* is the corresponding COX coefficient of the genes.

### 2.4. Tissue Sample Collection

ESCC tissues were obtained from the Department of Oncological Surgery of the Central Hospital of Shantou City, Guangdong Province, P.R. China, during 2012-2013 [22]. Tissues were collected from 121 ESCCs (Table 1) and confirmed by haematoxylin and eosin staining. This study was approved by the Ethics Committee of the Central Hospital of Shantou City.

### 2.5. RNA Extraction, cDNA Synthesis, and qRT-PCR

Total RNA from cells and tissues was extracted with TRIzol (Life Technologies, USA) according to the manufacturer's protocol. The purity and concentration of RNA were determined by OD260/280 ratio using a NanoDrop spectrophotometer, cDNA was obtained from the total RNA using random hexamers, and real-time PCR was performed by using a SYBR® Premix Ex Taq™ kit (DRR037A, Takara). The primer sequences for CTTN (cortactin), GGH (gamma-glutamyl hydrolase), GPD2 (glycerol-3-phosphate dehydrogenase 2), PYROXD2 (pyridine nucleotide-disulphide oxidoreductase domain 2), SRC (non-receptor tyrosine kinase), and SYNJ2BP (synaptojanin 2 binding protein) for real-time RT-PCR are shown in Table 2, and *β*-actin was used for normalization. Quantitative RT-PCR (qRT-PCR) was repeated at least three times [23, 24].

### 2.6. Cell Culture and Small RNA Interference

Cell lines used in this study were previously described [25, 26]. In brief, KYSE150 and KYSE510 esophageal carcinoma cells were grown in RPMI 1640 medium (Thermo Fisher Scientific) supplemented with 10% fetal bovine serum (GIBCO), penicillin-G (100 units/mL), and streptomycin (100 *μ*g/mL). Cells were incubated at 37°C in 5% CO_2_.

In functional assays, KYSE150 and KYSE510 cells were seeded into plates and cultured for 12–24 h until 70–80% confluence. ESCC cells were transfected with 30 nM or 75 nM siRNA using Lipofectamine 3000 (Invitrogen) according to the manufacturer's instructions. Then, cells were cultured and used for further analysis. Short interfering RNAs for CTTN, GGH, GPD2, PYROXD2, SRC, and SYNJ2BP and a negative control (NC) siRNA were synthesized by GenePharma (Suzhou, Jiangsu, China). The siRNA sequences targeting the flavoprotein signature are described in Table 2.

### 2.7. Colony Formation Assay

A colony formation assay was performed as described previously [27]. In brief, transfected cells were trypsinized and counted with a cell counter (Bio-Rad, Hercules, CA, USA). 1000–2000 cells per well were plated in a 6-well plate and incubated for 14 days at 37°C with 5% CO_2_. After washing twice with 4°C precooled PBS, cultures were fixed with ice-cold methanol for 20 min and stained with haematoxylin for 15 min. Colonies were photographed using ChemiDoc Touch (Bio-Rad). Each experiment was performed in triplicate.

### 2.8. Wound Healing Assay

KYSE150 and KYSE510 cells were transfected with siRNAs targeting the flavoprotein signature, and then cells were starved in serum-free medium for 12 h after being transfected for 36 h. Circles 3 mm in diameter were marked on the bottom of each dish to identify the areas for image capture and ensure that measurements were taken at the same locations. A wound was made by scraping the cell monolayer with a 200 *μ*L pipette tip. ESCC cells were maintained in RPMI 1640 medium with 2.5% fetal bovine serum. Images were captured at 0 and 36 h using a Leica DMI3000B inverted phase-contrast microscope (Leica Microsystems GmbH, Wetzlar, Germany). The wound closure rate was calculated from 6 images, using Image J (National Institutes of Health, Bethesda) analysis. Each experiment was performed in triplicate.

### 2.9. Transwell Assay

The transwell assay was performed as described previously [28]. KYSE150 and KYSE510 cells were starved in serum-free medium for 12 h after being transfected. A total of 5 × 10^4^ cells were plated in medium without serum in the upper well of a transwell chamber of a 24-well transwell with 8 *μ*m pores (BD Biosciences), which was placed in a bottom chamber containing medium supplemented with 10% fetal bovine serum. After 48 h, the membranes were fixed and stained with haematoxylin solution and scraped off the cells remaining in the upper chamber. The migration was quantified by counting 10 random fields under a Leica DMI3000B inverted phase-contrast microscope (400) with Image J. Each experiment was performed in triplicate.

### 2.10. Statistical Analysis

In training datasets, 179 ESCC patients were divided into low-risk (risk score ≤12.86) and high-risk groups (risk score >12.86) by using the median as a cutoff value [3]. In the independent validation datasets, 121 ESCC patients were divided into low-risk (risk score ≤1.28) and high-risk (risk score >1.28) groups by using the X-tile software [29]. Kaplan–Meier analysis was performed to test the survival distributions in the different groups. The sensitivity and specificity of the risk score for survival were compared by the receiver operating characteristic (ROC) curve [30]. All analyses were performed using the R program (www.r-project.org), including packages named pROC, survival, and randomForestSRC downloaded from Bio-conductor (http://www.bioconductor.org/). SPSS v13.0 software was used for statistical analysis. Where indicated, statistical analysis was performed by calculating means and SD. Graphs were mainly made by GraphPad Prism 6 (GraphPad, San Diego, USA). Differences between groups were evaluated with Student's *t* test. Significance was defined as *P* < 0.05. Gene set enrichment analysis (GSEA) was performed by the GSEA software [31].

## 3. Results

### 3.1. Acquisition of Flavoproteins and Their Interacting Protein Expression Level via Re-Annotation of the Agilent Array

179 ESCC samples (contained tumor tissues and adjacent normal tissues) from the GSE53625 dataset were selected according to the dataset screening criteria described in the Materials and Methods section. Several PCGs were identified by probe re-annotation of the Agilent-038314 CBC Homo sapiens lncRNA + mRNA microarray V2.0. We generated a new mRNA expression profile of the GSE53625 profiles by the following steps: First, we keep the probes which were mapped to the genomic coordinates of mRNAs uniquely. Second, we use the arithmetic mean to integrate the values of multiple probes mapping to the same mRNAs. Then, we identified 17434 PCGs in the dataset.

### 3.2. Selection of Flavoproteins and Interacting Protein Combinations for the Prognostic Signature in the Training Dataset

**Figure 1:**
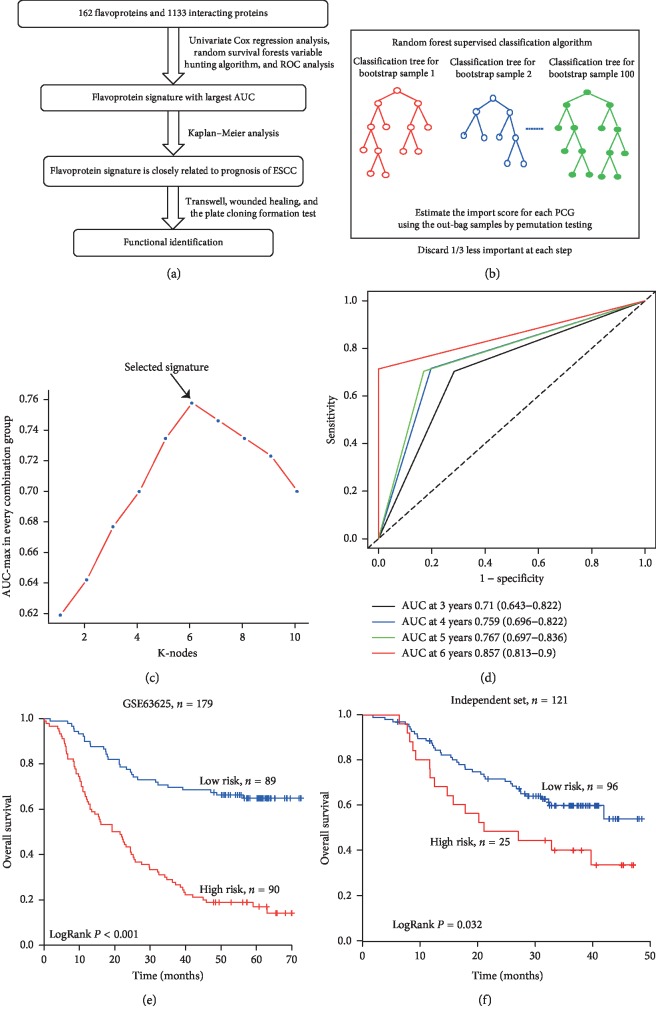
Identification of a flavoprotein signature predictive of overall survival of patients with ESCC. (a) Schematic diagram of the study. (b) Random forest supervised classification algorithm. (c) Procedure for identifying the final signature. The accuracies of all 1023 signatures were calculated, and the nine highest accuracies for *k* = 1, 2,…, 10 are shown in the plot and ROC for the flavoprotein signature prognostic model in the training dataset. (d) Time-dependent ROC for the flavoprotein signature prognostic model at 3–6 years in the training dataset. (e, f) Kaplan–Meier survival curves of patients classified into high- and low-risk groups, using the flavoprotein signature in the training and independent validation datasets. *P* values were calculated by the log-rank test.

The experimental process is shown in Figure 1(a). We extracted 162 flavoproteins and 1133 interacting proteins from the protein database. The ESCC patient cohort with 179 patients of ESCC was downloaded from the GEO database (GSE53625) and was used to explore the correlation between 1295 flavoproteins and their corresponding interacting proteins. Firstly, the univariate Cox proportional hazards regression analysis identified a flavoprotein family set composed of 6 flavoproteins and 47 interacting proteins, which were associated with overall survival (*P* < 0.05), to serve as prognostic genes. Secondly, ten genes most related to the prognostic classification were selected among the flavoprotein family set according to the permutation importance score by using random survival forests variable hunting (RSFVH) algorithm (Figure 1(b)).

We compared the risk-score model of 2^10^−1 = 1023 combinations of the flavoprotein set by the ROC curve to elect a better prediction prognostic signature. All the risk scores of the flavoprotein signature are described in the Materials and Methods section. Then, the flavoprotein signature with the max AUC is selected. The signature was composed of two flavoprotein genes (GPD2 and PYROXD2) and four flavoprotein interacting protein genes (CTTN, GGH, SRC, and SYNJ2BP), and the risk score was obtained as follows: risk score = (−0.77 × expression level of GPD2) + (0.28 × expression level PYROXD2) + (0.33 × expression level of CTTN) + (−0.39 × expression level of GGH) + (0.54 × expression level of SRC) + (0.48 × expression level of SYNJ2BP). AUC of the flavoprotein signature in the prognostic model was 0.76 for survival status (Figure 1(c), Table 3), and time ROC analysis showed that AUC of the signature is 0.710 (95% CI: 0.643–0.822) at 3 years, 0.759 (95% CI: 0.696–0.822) at 4 years, 0.767 (95% CI: 0.697–0.836) at 5 years, and 0.857 (95% CI: 0.813–0.900) at 6 years (Figure 1(d)).

### 3.3. The Flavoprotein Signature Could Predict ESCC Patients' Survival in Training Dataset and Independent Validation Datasets

By using the median risk score of the flavoprotein signature as the cutoff point, 179 ESCC patients of the training dataset were divided into the high-risk group (*n* = 90) and low-risk group (*n* = 89). The high-risk group had a significantly shorter OS than the low-risk group (*P* < 0.001, mean survival time: 26.77 months vs. 54.97 months; Figure 1(e)).

To confirm the findings described above, we also evaluated the efficiency of the constructed expression-defined flavoprotein prognostic model in independent validation datasets (*n* = 121). The same flavoprotein model was used to calculate the flavoprotein signature-based risk scores for 121 patients in this dataset.  Figure 1(f) shows the Kaplan–Meier curves of the model in the validation datasets (*P*=0.032, mean survival time: 15.73 months vs. 21.1 months).

### 3.4. Knocking Down the Flavoprotein Signature Components in ESCC Cell Lines Affects Cell Migration and Growth

**Figure 2:**
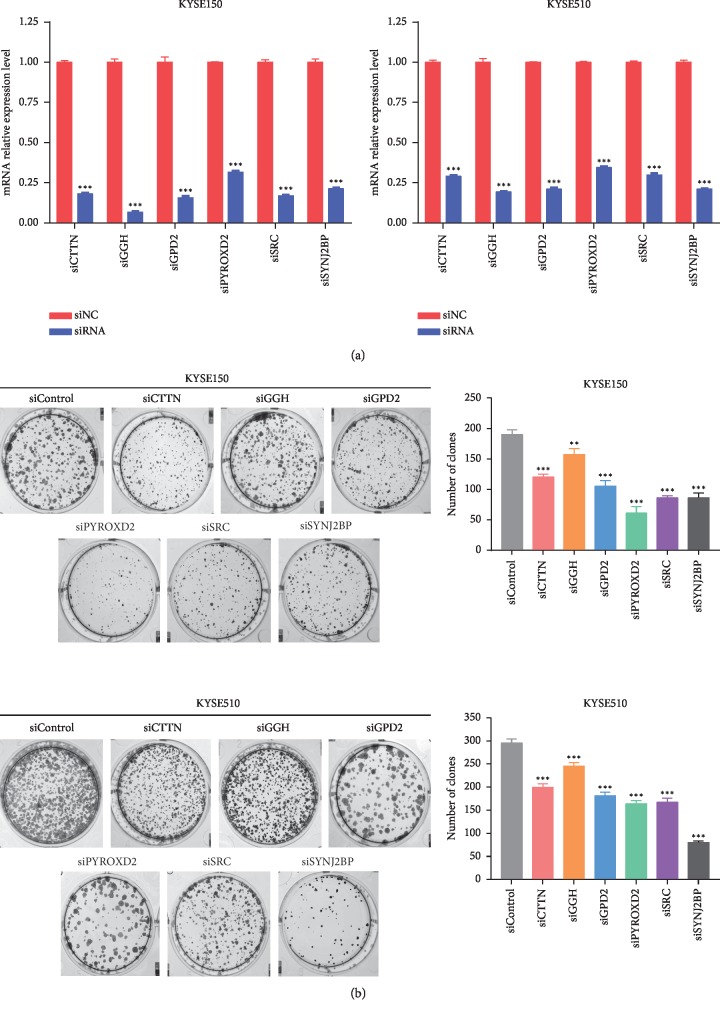
Knockdown of the flavoprotein signature inhibits proliferation of ESCC cells. (a) siRNA-mediated knockdown of the flavoprotein signature was examined by using qRT-PCR. *β*-Actin served as the loading control. Negative control (NC) siRNA or siRNA targeting the flavoprotein signature (siRNA) was transfected into KYSE150 cells and KYSE510 cells. ^*∗*^*P* < 0.05, ^*∗∗*^*P* < 0.01, ^*∗∗∗*^*P* < 0.001, Student's *t* test. (b). Clone formation images and number of clones. Cell proliferation was determined in colony formation assays in which 2000 transfected cells were inoculated in each well of a six-well plate. Cultures were maintained for 2 weeks, and cells were then fixed, stained, and photographed. Each experiment was performed in triplicate, and results represent the mean ± SD of three experiments. ^*∗*^*P* < 0.05, ^*∗∗*^*P* < 0.01, ^*∗∗∗*^*P* < 0.001, one-way ANOVA.

**Figure 3:**
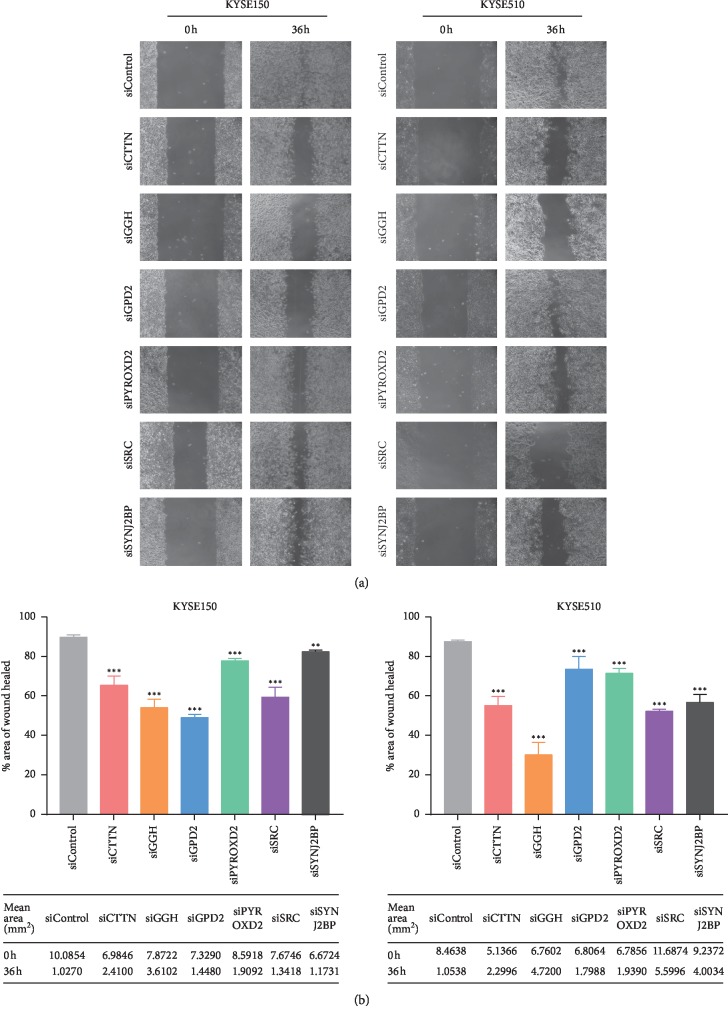
Knockdown of flavoprotein signature components reduces cell migration in a wound healing assay. (a). Wound healing images in KYSE150 and KYSE510 cells. (b) Top: rate of wound closure of KYSE150 and KYSE510 cells following transfection of siRNA and siNC. For quantification, the cells were counted in 6 random fields under a light microscope (×400). Data represent the mean ± SD of triplicate. ^*∗*^*P* < 0.05, ^*∗∗*^*P* < 0.01, ^*∗∗∗*^*P* < 0.001, one-way ANOVA. Bottom: mean wound area (mm^2^) at 0 hours and 36 hours.

**Figure 4:**
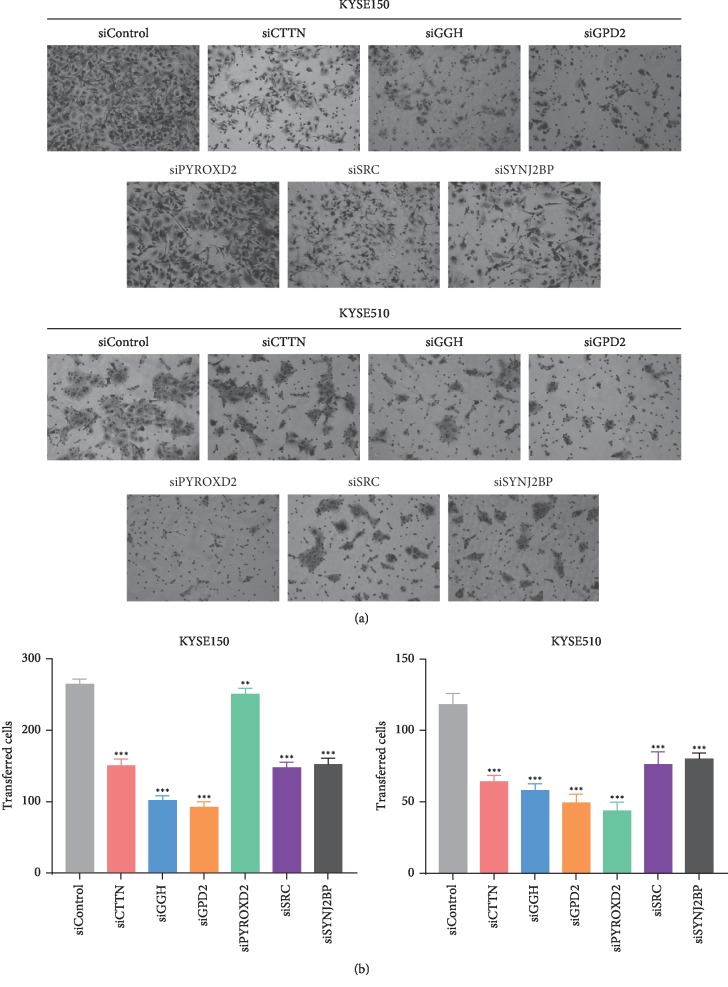
Knockdown of flavoprotein signature components reduces cell migration in a transwell assay. (a) Migratory images of KYSE150 and KYSE510 cells. (b) A transwell assay was used to determine the effects of siRNA-mediated knockdown of flavoprotein signature components on cell migration. Migrating cells were fixed and stained, and representative fields were photographed. For quantification, cells were counted in 10 random fields under a light microscope (×400). Data represent mean ± SD of triplicate. ^*∗*^*P* < 0.05, ^*∗∗*^*P* < 0.01, ^*∗∗∗*^*P* < 0.001; one-way ANOVA.

To assess the effect of the flavoprotein signature expression in ESCC cells, siRNAs targeting the flavoprotein signature genes were transfected into KYSE510 cells and KYSE150 cells, and the silencing of the flavoprotein signature was determined by qRT-PCR.  Figure 2(a) shows that the relative mRNA expression of the flavoprotein signature in KYSE150 cells and KYSE510 cells in the si-flavoprotein signature group was lower than the si-negative control (NC) group (*P* < 0.01). Colony formation assay showed that knockdown of the flavoprotein signature genes significantly inhibited the growth of KYSE150 cells and KYSE510 cells (Figure 2(b)). Compared with the siNC group, ESCC cell growth in the si-flavoprotein signature group was generally suppressed (*P* < 0.0001, one-way ANOVA). Migration of ESCC cells was examined in each group by wound healing and transwell assays. As shown in Figures 3 and 4, siRNA-mediated knockdown of CTTN, GGH, GPD2, PYROXD2, SRC, or SYNJ2BP in KYSE150 cells and KYSE510 cells conferred reduced migration, compared with the siNC group (*P* < 0.01, one-way ANOVA). However, six genes in the flavoprotein signature produce different degrees of effect on ESCC cell proliferation and migration. And six genes also play different roles in KYSE150 and KYSE510 cells. After knocking down the PYROXD2 gene in KYSE150 cells, the cell proliferation ability was most significantly reduced (*P* < 0.0001, one-way ANOVA), while wound healing and transwell assay showed that knockdown of the PYROXD2 gene in KYSE150 cells had the least effect on cell migration compared to other genes (*P* < 0.01, one-way ANOVA). In KYSE510 cells, the significant effect of inhibiting cell proliferation is knocking down the SYNJ2BP gene (*P* < 0.0001, one-way ANOVA). In KYSE150 and KYSE510 cells, the effect of GGH on cell proliferation was minimal compared to other genes (*P* < 0.01, one-way ANOVA). However, after knockdown of GGH, wound healing and transwell assay showed that the GGH gene has a great influence on the mobility of ESCC cells (*P* < 0.0001, one-way ANOVA). These results imply that the flavoprotein signature plays important but different roles in the progression of ESCC.

### 3.5. Functional Characterization of the Flavoprotein Signature

**Figure 5:**
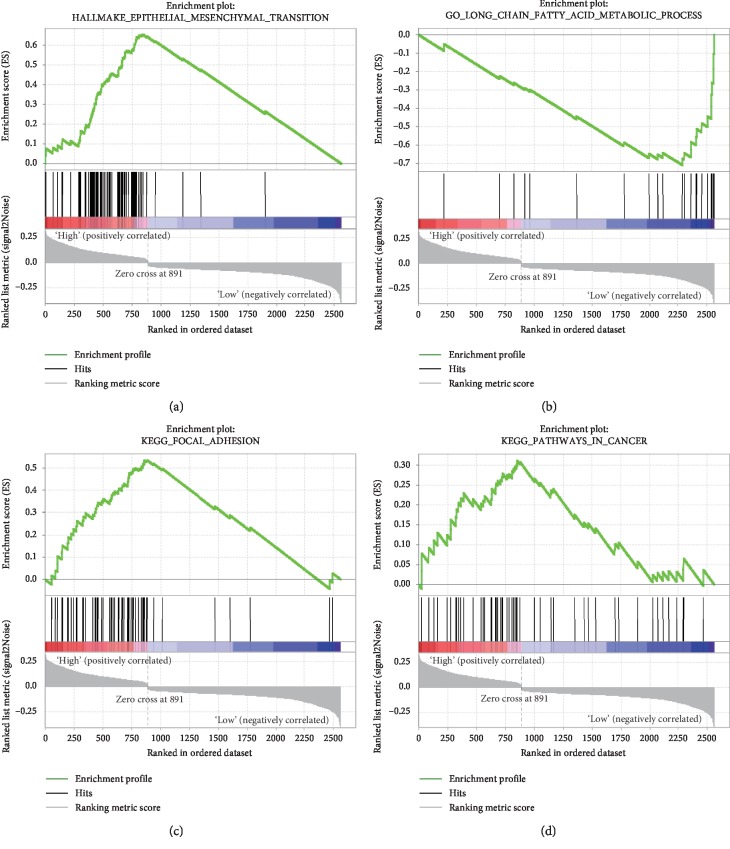
Predicting the function of genes in the signature. Gene set enrichment analysis (GSEA) showed that the flavoprotein signature was significantly associated with EMT pathways and long chain fatty acid metabolism (a, b). Two KEGG pathways (focal adhesion and cancer-related pathways) were significantly enriched in the enriched gene sets (c, d) (*P* < 0.05, FDR < 0.05).

To further explore the potential biological function of this signature, we compared the gene expression profiles of ESCC patients classified as high-risk and low-risk by the flavoprotein signature in the training set (GSE53625). The gene sets with significantly different expression (FDR < 0.05) between high-risk and low-risk were selected for gene set enrichment analysis (GSEA). Several clusters of genes functionally related to more than 150 GO terms and 20 KEGG pathways were observed. These data suggest that the flavoprotein signature might affect tumorigenesis and development through interacting with many important biological processes, such as epithelial mesenchymal transition, focal adhesion, oxidative phosphorylation, and long chain fatty acid metabolism (Figure 5).

## 4. Discussion

Esophageal cancer is the fifth most common and fourth most lethal malignant tumor in China [1]. Currently, the therapeutic efficacy of treatment is quite limited, with patients exhibiting a low five-year incidence of survival. In recent years, many reports have used gene arrays to analyze the gene expression profiles and predict prognostic signature in esophageal cancer. So far, however, there are no clinical predictive markers specific to the early diagnosis of ESCC. Therefore, the identification and validation of new novel biomarkers have vital significance in the diagnosis and treatment of esophageal cancer. Increasing evidences indicate that flavoproteins and their interacting proteins are involved in tumorigenesis and may serve as potential biomarkers [32, 33]. In this paper, we explored the clinical significance of flavoproteins (GPD2 and PYROXD2) and their interacting proteins (CTTN, GGH, SRC, and SYNJ2BP). The signature composed of six members is not only involved in proliferation and migration of ESCC cells but also related with OS of ESCC patients. Furthermore, the combination of CTTN, GGH, GPD2, PYROXD2, SRC, and SYNJ2BP can accurately predict the prognosis of ESCC patients, with accuracies for predicting 6-year OS for ESCC patients (AUC = 0.857, 95% CI: 0.813–0.9). The signature composed of flavoproteins and their interacting proteins shows prognostic power in ESCC patients.

In this study, we used different statistics and machine learning methods to identify an expression signature, involving flavoproteins and their interacting proteins, that is associated with survival of ESCC patients. The six genes CTTN, GGH, GPD2, PYROXD2, SRC, and SYNJ2BP with the largest AUC were selected as the flavoprotein signature. CTTN and SRC are two of the most studied oncogenes. SRC is a non-receptor protein tyrosine kinase that is activated following engagement of many different classes of cellular receptors [34]. CTTN is a major substrate of the SRC tyrosine kinase and contributes to the organization of the actin cytoskeleton and cell shape [35]. CTTN and SRC have been implicated in cell proliferation, motility, and invasion in various types of cancer, such as esophageal cancer, colorectal cancer, laryngeal carcinoma, and lung cancer [36–41]. GPD2 and PYROXD2 are both flavoproteins that contain noncovalently bound FAD as cofactor. In later years, researchers found GPD2 is the target gene for many diseases, such as febrile seizures, nonspecific mental retardation, and diabetes [42–44]. Moreover, a study in Canada suggested that GPD2 can be a target for cancer therapeutics [45]. PYROXD2 is pyridine nucleotide-disulfide oxidoreductase domain 2 with oxidoreductase activity [46]. Montoliu et al. confirmed the effect of PYROXD2 polymorphisms on trimethylamine metabolism [47]. Hong et al. found that PYROXD2 can be a target gene for prostate cancer [48]. GGH plays an important role in the metabolism of pteroylpolyglutamates and antifolates [49]. Many reports have shown that GGH is involved in the ERG-negative prostate cancer and gastric cancer development by multiple methods [50, 51]. SYNJ2BP regulates endocytosis of activin type 2 receptor kinases through the Ral/RALBP1-dependent pathway. Liu et al. confirmed that SYNJ2BP influences tumor growth and metastasis by activating the DLL4 pathway in hepatocellular carcinoma [52]. SYNJ2BP also plays an important role in breast cancer and renal cell carcinoma metastasis [53, 54]. Although a series of previous articles have revealed the potential value of flavoproteins and their interacting proteins in cancer prognosis prediction, such as CTTN, SRC, and GPD2, using the combination of flavoproteins and their interacting proteins in predicting ESCC prognosis has not been elucidated clearly. Here, we analyzed the mRNA expression profiles of patients with ESCC downloaded from GEO and applied the RSFVH algorithm and ROC to pick out flavoproteins and their interacting proteins and reduce the high dimension. Next, we identified a signature including several flavoproteins and their interacting proteins, which are strongly associated with the overall survival. Then, we constructed time-dependent ROC curves to assess the sensitivity and specificity of variables and calculated the corresponding AUC.

Functionally, we knocked down the flavoprotein signature by transfecting siRNA into ESCC cells. Next, the functionally well-defined CTTN and SRC were used as positive controls to compare the effect of the remaining four genes on proliferation and migration of transfected ESCC cells. Similar to knockdown of CTTN and SRC, ESCC cell growth and motility are significantly reduced following knockdown of GGH, GPD2, PYROXD2, and SYNJ2BP. Among these genes, GPD2 knockdown has the most significant effect on inhibiting the proliferation and migration of ESCC cells. The GPD2 gene encodes the mitochondrial glycerol-3-phosphate dehydrogenase, which is localized to the outer surface of the inner mitochondrial membrane. Mitochondrial glycerol-3-phosphate dehydrogenase, as a component of the glycerophosphate shuttle, functions at the crossroads of glycolysis, oxidative phosphorylation, and fatty acid metabolism. Mitochondrial glycerol-3-phosphate dehydrogenase regulates both the glycerol-3-phosphate and malate-aspartate shuttles, which play important roles in tumor metabolism [55–58]. GSEA analyses also suggest that the flavoprotein signature mainly affects the function of tumor cells by affecting metabolic pathways. The precise role played by GPD2 in ESCC needs further study. This study still has many limitations. For example, we only studied the flavoprotein signature as a whole, but we did not learn the mechanism of one or several genes. In addition, our study only stayed at the RNA level, but not at the protein level. Other aspects will be considered in future research.

In summary, this is the first study to investigate a signature, comprised of flavoproteins and their interacting proteins, in patients with esophageal squamous cell carcinoma. Furthermore, abnormal expression of the flavoprotein signature promotes proliferation and migration of ESCC cells. These results implicate components of the flavoprotein signature as efficient prognostic markers and potential targets in gene therapy for ESCC.

## Figures and Tables

**Table 1:** Clinical characteristics of 121 ESCC patients used for validation experiments.

Clinical parameters	Number
Total cases	121
Status	
Deceased	54
Living	67
Mean age, year	57.8
Age	
<57.8	62
≥57.8	59
Gender	
Male	92
Female	29
pTNM stage	
I	6
II	57
III	58

**Table 2:** Primer sequences for qRT-PCR and siRNA sequences.

Gene	Sequence (5′-3′)
	Primers for qRT-PCR
CTTN-qF	AACGATCTGGGGATCACAGC
CTTN-qR	CCAGCCGTCGTCAATCATC T
PXROXD2-qF	CAAGCTCAGCCACCACACAT
PXROXD2-qR	GCTTCTCACCTCTGTGCCAT
SYNJ2BP-qF	CTGCACCAGGATGCTGTAGAC
SYNJ2BP-qR	GAAAGCCCAGGCTGCTACCAT
GGH-qF	GATGGCATTTCCCATGCACC
GGH-qR	TGCTTTCTCCTCTTCAGATTCAG
GPD2-qF	GTGGCCAAAATGGCAAGTGT
GPD2-qR	AATCCTGGGTAGGGCTTCCT
SRC-qF	GTGGGAGAGAACCTGGTGTG
SRC-qR	GATGGTGAAGCGGCCATAGA
ACTB-qF	CAACTGGGACGACATGGAGAAA
ACTB-qR	GATAGCAACGTACATGGCTGGG
	siRNA target sequence
CTTN-Homo-449	CCAUGGCUAUGGAGGGAAATT
PYROXD2-Homo-1328	CCUCCUUCAUCAGGCCUUUTT
SYNJ2BP-Homo-2746	GGACAAGUUGAAGACCCUUTT
GGH-Homo-338	GCGAGCCUCGAGCUGUCUATT
GPD2-Homo-494	GCAUUUCAGAACCAGUUAATT
SRC-Homo-937	GCCUCUCAGUGUCUGACUUTT
Negative control	UUCUCCGAACGUGUCACGUTT

**Table 3:** Identities of flavoproteins and their interacting proteins in the prognostic expression signature and their univariate Cox association with prognosis.

Ensembl ID	Gene symbol	Gene description	Coefficient^a^	*P* value^a^	Gene expression level association with prognosis	Chromosome location
ENSG00000115159	GPD2	Glycerol-3-phosphate dehydrogenase 2	−0.77	0.01	Low	2 : 156435290–156613735 : 1
ENSG00000119943	PYROXD2	Pyridine nucleotide-disulphide oxidoreductase domain 2	0.28	0.00	High	10 : 98383565–98415184 : −1
ENSG00000085733	CTTN	Cortactin	0.33	0.00	High	11 : 70398404–70436584 : 1
ENSG00000137563	GGH	Gamma-glutamyl hydrolase	−0.39	0.01	Low	8 : 63015079–63039171 : −1
ENSG00000197122	SRC	Non-receptor tyrosine kinase	0.54	0.02	High	20 : 37344685–37406050 : 1
ENSG00000213463	SYNJ2BP	Synaptojanin 2 binding protein	0.48	0.02	High	14 : 70366496–70417061 : −1

^a^Derived from the univariable Cox regression analysis in the training set.

## Data Availability

No data were used to support this study.

## Abbreviations

AUC: Area under the curve
CI: Confidence interval
CTTN: Cortactin
ESCC: Esophageal squamous cell carcinoma
GEO: Gene Expression Omnibus
GGH: Gamma-glutamyl hydrolase
GO: Gene ontology
GPD2: Glycerol-3-phosphate dehydrogenase 2
HR: Hazard ratio
KEGG: Kyoto Encyclopedia of Genes and Genomes
OS: Overall survival
PCG: Protein coding gene
PYROXD2: Pyridine nucleotide-disulphide oxidoreductase domain 2
ROC: Receiver operating characteristic
SD: Standard deviation
SRC: Non-receptor tyrosine kinase
SYNJ2BP: Synaptojanin 2 binding protein.
